# The Parasitic Plant *Cuscuta australis* Is Highly Insensitive to Abscisic Acid-Induced Suppression of Hypocotyl Elongation and Seed Germination

**DOI:** 10.1371/journal.pone.0135197

**Published:** 2015-08-10

**Authors:** Juan Li, Christian Hettenhausen, Guiling Sun, Huifu Zhuang, Jian-Hong Li, Jianqiang Wu

**Affiliations:** 1 College of Plant Science and Technology, Huazhong Agricultural University, Wuhan, Hubei, China; 2 Key Laboratory of Economic Plants and Biotechnology, Kunming Institute of Botany, Chinese Academy of Sciences, Kunming, Yunnan, China; 3 Yunnan Key Laboratory for Wild Plant Resources, Kunming Institute of Botany, Chinese Academy of Sciences, Kunming, Yunnan, China; Henan Agricultural Univerisity, CHINA

## Abstract

Around 1% of angiosperms are parasitic plants. Their growth and development solely or partly depend on host plants from which they extract water, nutrients, and other molecules using a parasitic plant-specific organ, the haustorium. Strong depletion of nutrients can result in serious growth retardation and in some cases, death of the hosts. The genus *Cuscuta* (dodder) comprises about 200 holoparasitic species occurring on all continents. Their seedlings have no roots and cotyledons but are only string-like hypocotyls. When they contact suitable host plants, haustoria are formed and thereafter seedlings rapidly develop into vigorously growing branches without roots and leaves. This highly specialized lifestyle suggests that *Cuscuta* plants likely have unique physiology in development and stress responses. Using germination and seedling growth assays, we show that *C*. *australis* seeds and seedlings are highly insensitive to abscisic acid (ABA). Transcriptome analysis and protein sequence alignment with Arabidopsis, tomato, and rice homologs revealed that *C*. *australis* most likely consists of only four functional ABA receptors. Given that *Cuscuta* plants are no longer severely challenged by drought stress, we hypothesize that the ABA-mediated drought resistance pathway in *Cuscuta* spp. might have had degenerated over time during evolution.

## Introduction

Parasites are defined as organisms that obtain nutrients from hosts and cause harm but not immediate death [1]. Plant parasitism is thought to have evolved independently at least 12 or 13 times and it was estimated that about 1% of angiosperm plants are parasitic [2]. Compared with autotrophic plants, parasitic plants have unique physiology, ecology, and evolution. Using a special organ, haustorium, which penetrates the host tissues and forms a connection between the host and parasite, parasitic plants draw nutrients and other molecules from hosts. The growth and development of parasitic plants are solely or partly dependent on the hosts.

The plants in the genus *Cuscuta* (dodder) are shoot obligate parasites. This genus consists of about 200 species that occur on all continents [3], and is known to have a wide variety of hosts, including crops and wild plants. *Cuscuta* not only extracts small molecules from hosts, such as sugars and amino acids, but also large molecules: GFP (green fluorescence protein) from GFP-expressing tobacco is translocated to *Cuscuta*, indicating symplastic transport of proteins [4]. Moreover, *Cuscuta* also transports mRNAs from pumpkin, Arabidopsis, and tomato [5, 6] and mRNAs could be transported as far as 20 cm away from the connecting point of *Cuscuta* and the host [7].

Unlike many other parasitic plants, *Cuscuta* has unique morphological properties. After germination, *Cuscuta* seedlings emerge with thread-shaped hypocotyls, and having no roots and cotyledons, they must find suitable hosts within a few days to survive. Contact between a *Cuscuta* seedling and a host often induces haustorium formation and this is the first step in establishing parasitism. After haustoria penetrate into host tissues and the vascular systems fuse, *Cuscuta* rapidly grows and develops many more branches, concomitantly with the formation of numerous haustoria. Resulting nutrient loss and decreased photosynthesis capacity can largely compromise growth and development of the host plants.

Many genetic and biochemical studies indicate that hormones play pivotal roles in regulating plant development and response to environmental stresses. For example, auxin is very important for embryogenesis, vascular differentiation, organogenesis, tropic growth, and formation of root and shoot architecture [8]. Compromised jasmonic acid (JA) biosynthesis or signaling leads to highly decreased defense against insects and necrotrophic fungi and also causes sterility [9]. Gibberellins (GAs) are important for stem and leaf growth, and in some species they functions in seed germination and the development of flowers, fruits, and seeds [10].

Abscisic acid (ABA) serves as an endogenous messenger in biotic and abiotic stress responses of plants [11–14]. It mediates seed dormancy, controls seedling development, and triggers tolerance to abiotic stresses, including drought [15, 16]. Drought stress induces strong increase of plant ABA levels, accompanied by a major change in gene expression and adaptive physiological responses [17–19], such as decreased stomatal aperture to reduce transpirational water loss [20]. Although the biosynthesis, signaling, and functions of phytohormones in autotrophic plants have been intensively studied, yet very little is known about the responses of parasitic plants to hormones. *Cuscuta australis* R.Br is native in South China [21], and it contains very little chlorophyll [22] and solely relies on obtaining photosynthetic assimilates, water, and other molecules from the hosts [23, 24]. Using germination and seedling growth assays, we examined the responses of *C*. *australis* to ABA. Remarkably, germination and seedling assays indicated that *C*. *australis* was almost completely insensitive to ABA. Furthermore, transcriptome data-mining suggested that *C*. *australis* likely had only 4 functional ABA receptors and this is much less than those in Arabidopsis, tomato, and rice. These data suggest that *C*. *australis* has a distinct ABA hormonal physiology, and we propose that the highly adapted and specialized lifestyle may have relaxed the selection pressure from drought stress in *C*. *australis* and have led to loss of responses to ABA.

## Materials and Methods

### Seed germination and seedling growth assays

*Cuscuta australis* were propagated by parasitizing on soybean (*Glycine max* (L) Merrill). To break dormancy, *C*. *australis* seeds were soaked in concentrated sulfuric acid for 0.5 h and subsequently washed with distilled water for at least 5 times. Tomato (*Solanum lycopersicum* cv. White Fruit Qiangfeng) seeds were purchased from HEBEIJINGDESEED CO., LTD and were surface-sterilized for 2 min in 75% ethanol, followed by 5 min incubation in 3% sodium hypochlorite solution and 5 washes with sterile distilled water. To determine ABA-dependent germination inhibition, *C*. *australis* and tomato seeds were sown on solid 1/2 MS medium with different concentrations of ABA and incubated in a growth chamber (24°C, 16 h light/8 h dark cycle). The germination rate was monitored daily.

For seedling grow assays, seeds were allowed to germinate on 1/2 MS medium. When seedlings reached the size of about 1cm, they were transferred to individual sterile glass tubes containing liquid 1/2 MS medium with or without different concentration of ABA and incubated in a growth chamber (24°C, 16 h light/8 h dark cycle). Five days after the transfer, *C*. *australis* and tomato seedlings were photographed and their lengths were recorded. For all treatments 10 replicates were used and the experiment was repeated 3 times.

### Drought treatment

*C*. *australis* and tomato seeds were germinated on 1/2 MS medium and allowed to grow until plants reached a size of 3–5 cm. Seedlings were placed on a plastic foil to air dry at 24°C, and 5 to 10 seedlings (about 0.1 g) were pooled to form 1 replicate, and the dehydration status was recorded over a period of 24 h. When 30, 50, or 70% water loss was reached, samples were taken, frozen in liquid nitrogen and stored at -80°C until analysis.

### Extraction and quantification of ABA

High performance liquid chromatography-tandem mass spectroscopy (HPLC-MS/MS) was used to measure the levels of ABA [25]. Samples (~100 mg of fresh mass) were ground in liquid nitrogen and 1 ml of ethyl acetate spiked with 20 ng of the internal standard (^2^H_6_)-ABA was added. After a 10 min vortexing step, followed by centrifugation at 13,000 *g* for 15 min at 4°C, the organic supernatants were evaporated to dryness in a vacuum concentrator (Eppendorf, Hamburg, Germany) at 30°C. Samples were resuspended in 600 μl of a methanol: water (70:30, v/v) solution and again centrifuged to remove particles. The supernatants were analyzed on a Shimadzu 8040 HPLC-MS/MS system.

### Statistical analysis

The significance of the differences between control and treated groups was analyzed using Student’s *t*-test. Asterisks indicate significant differences between the control and treated groups (*, P < 0.05; **, P < 0.01; ***, P < 0.001). Data were expressed as mean ± SE (standard error).

### Phylogenetic analysis

Fourteen *A*. *thaliana* ABA receptors *PYR/PYL/ RCAR* have been annotated in the NCBI database (http://www.ncbi.nlm.nih.gov/). To identify the ABA receptors in *C*. *australis*, the 14 Arabidopsis *PYR/PYL/ RCAR* amino acid sequences were used as queries to blast against a homemade transcriptome assembly of *C*. *australis*. This homemade transcriptome database was constructed from seeds, just germinated seeds, seedlings, pre-haustoria, flower buds, flowers and capsules, and was finally assembled from 472,413,806 clean reads. The 4 putative *C*. *australis* ABA receptor genes that we identified have been deposited in the NCBI under accession numbers: KR232648 to KR232651. Twelve ABA receptors in rice (*Oryza sativa*) (*OsPYL1* to *OSPYL12*) have been annotated by the MSU Rice Genome Annotation Project (http://rice.plantbiology.msu.edu/) [26], and 15 ABA receptors in tomato (*Solanum lycopersicum*) have been deposited in the Phytozome database (http://www.phytozome.net) [27]. The phylogenetic tree was generated using MEGA5 [28].

## Results

### The response of *C*. *australis* seeds and seedlings to ABA

*Cuscuta* seedlings are basically hypocotyls and mature plants have only stems (and flowers later). In both stages, these plants transpire very little and have almost no stomata (data not shown). In addition, *Cuscuta* hijacks the vascular systems of the host plants for water and the hosts usually have well-controlled water contents, even under drought conditions. Thus, it is possible that, since they evolved parasitism, *Cuscuta* plants are no longer strongly challenged by drought stress.

Exogenous phytohormone application to seedling has long been used to study hormone biosynthesis, signaling, and functions, and numerous mutants have been identified using this screening approach. Given the relatively large size of tomato seedlings, especially its long hypocotyl, we selected tomato to compare with *C*. *australis* and study the responses to exogenously applied ABA, the most important hormone in modulating drought responses.

**Fig 1:**
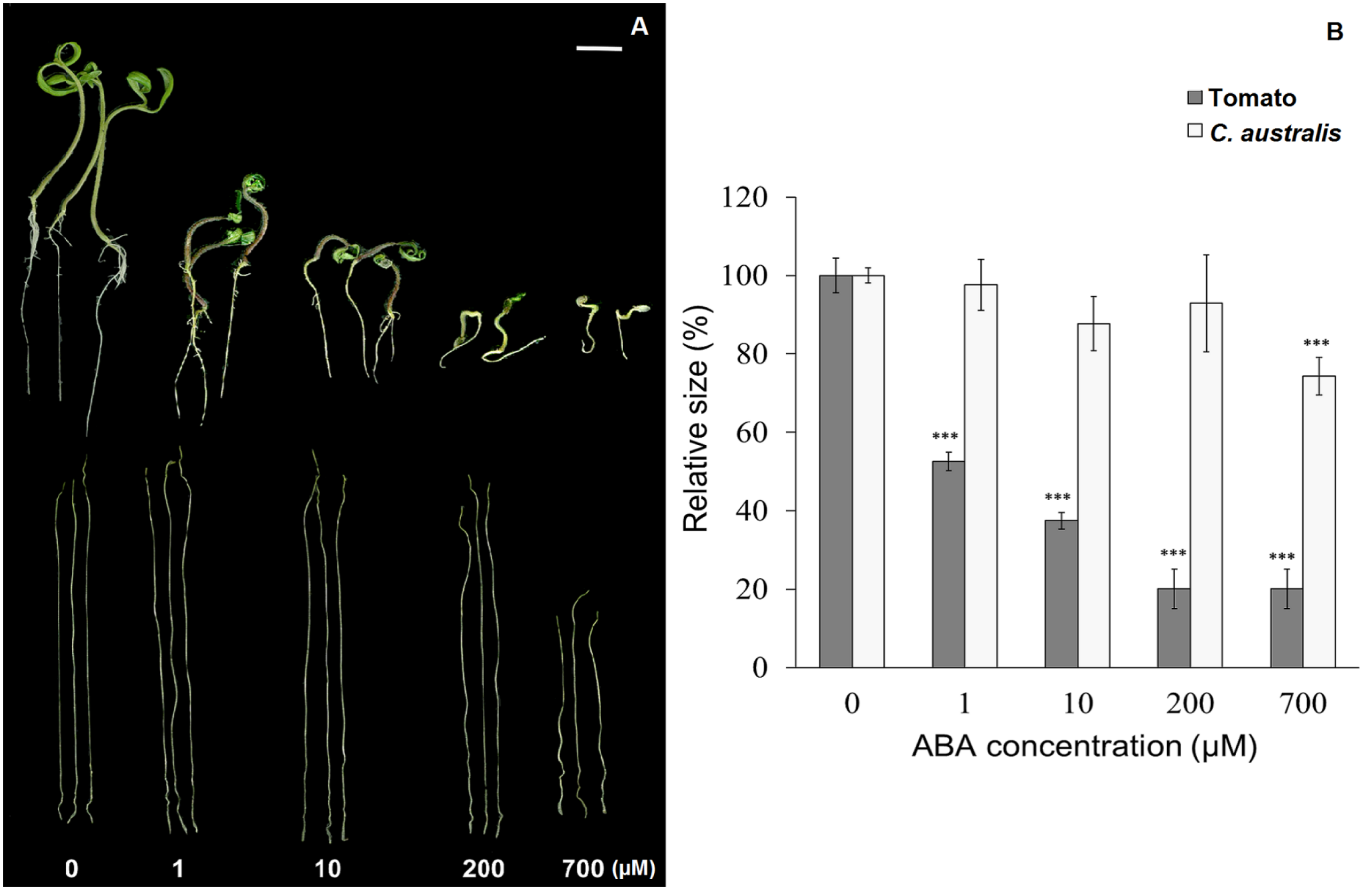
*C*. *australis* seedlings are highly insensitive to ABA. *C*. *australis* and tomato seeds were germinated on ½ MS plates, and when seedlings grew to 1 cm in length, they were transferred to liquid ½ MS medium containing different concentrations of ABA to grow for 5 more days. (**A**) A photograph of tomato (top) and *C*. *australis* seedlings (bottom) (Scale bar = 1cm). (**B**) The lengths of tomato hypocotyls and *C*. *australis* seedlings relative to those of the controls (without ABA). Results are presented as the mean ± SE (N = 10). Asterisks indicate significant differences between the respective control and treated groups determined by Student’s *t*-test (***, P < 0.001).

Tomato seedlings were sensitive to ABA treatment and their hypocotyl lengths were significantly reduced at a concentration of 1 μM ABA (not only hypocotyls but also roots were affected by the treatment) (Fig 1). In contrast, *C*. *australis* seedlings did not show growth inhibition at 1 μM of ABA and even up to 200 μM of ABA (a concentration at which tomato seedling had died already); only when 700 μM of ABA were applied, the lengths of *C*. *australis* seedlings were moderately reduced (25%) (Fig 1). Thus, *C*. *australis* seedlings were extremely hyposensitive to ABA-induced suppression of hypocotyl elongation.

**Fig 2:**
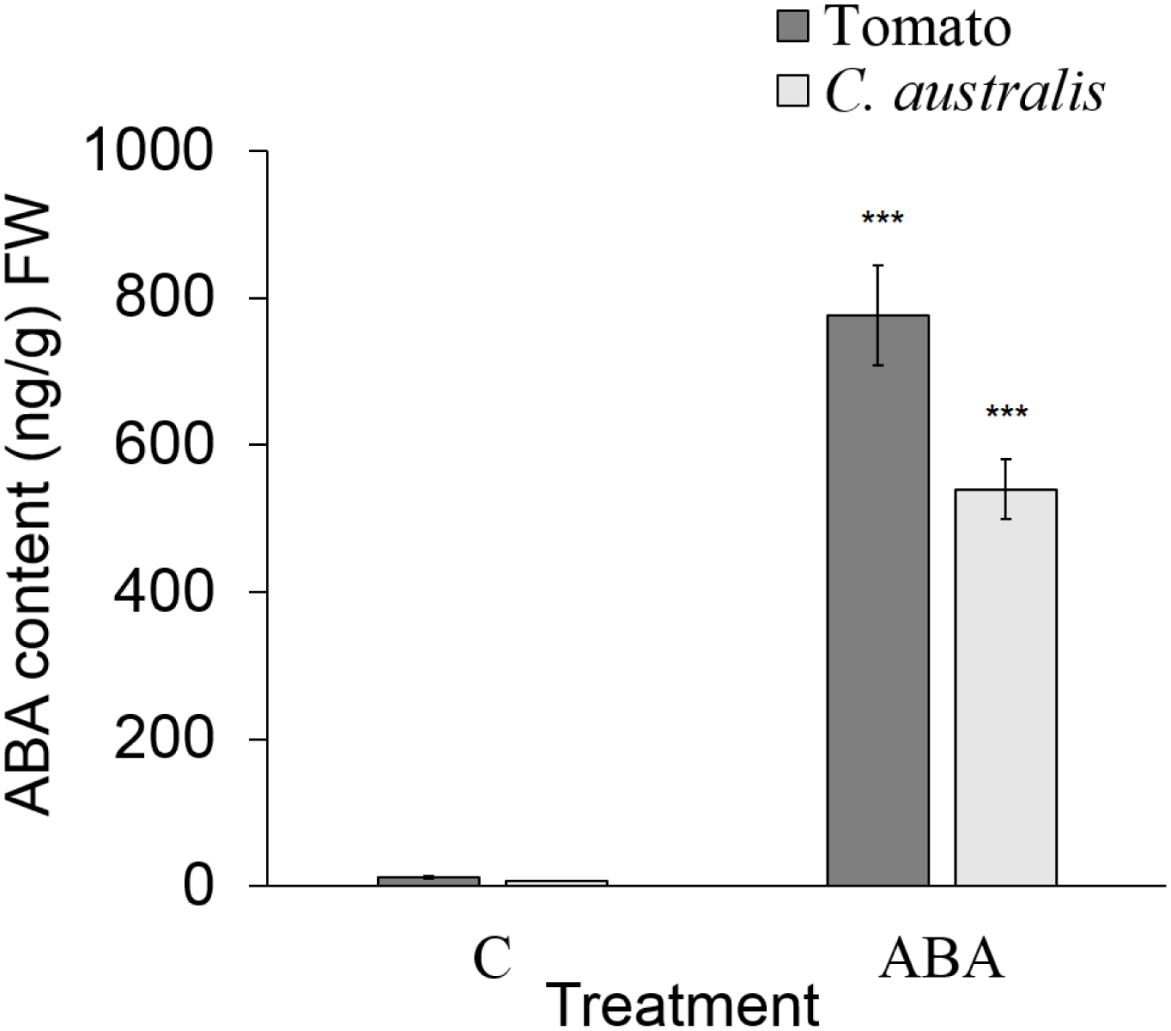
*C*. *australis* seedlings can take up ABA from the media. *C*. *australis* and tomato seedlings were germinated and grown to about 1cm in length before they were transferred to 1/2 MS medium containing 50 μM of ABA or no ABA (indicated by ABA and C on the X-axis) and incubated for 24 h. Subsequently, seedlings were thoroughly rinsed with water to remove ABA from the surface. Five to 10 seedlings (~100 mg) were pooled to form 1 replicate, and ABA levels were measured (mean ± SE; N = 5). Asterisks indicate significant differences between the respective ABA-treated and non-treated groups determined by Student’s *t*-test (***, P < 0.001).

Since *C*. *australis* is rootless, there is a possibility that ABA in the liquid medium could not enter the seedlings, and thus they showed no suppressed hypocotyl elongation response to ABA. In order to test this possibility, ABA contents in *C*. *australis* and tomato seedlings were measured after they were treated with 50 μM of ABA for 24 h. The ABA levels increased up to 776.5 ng/g FW in tomato and 539.6 ng/g FW in *C*. *australis* seedlings, while the water-treated controls had only 11.6 ng/g FW and 7.1 ng/g FW of ABA, respectively (Fig 2). Therefore, we ruled out the possibility that ABA cannot be diffused into or taken up by *C*. *australis* seedlings.

**Fig 3:**
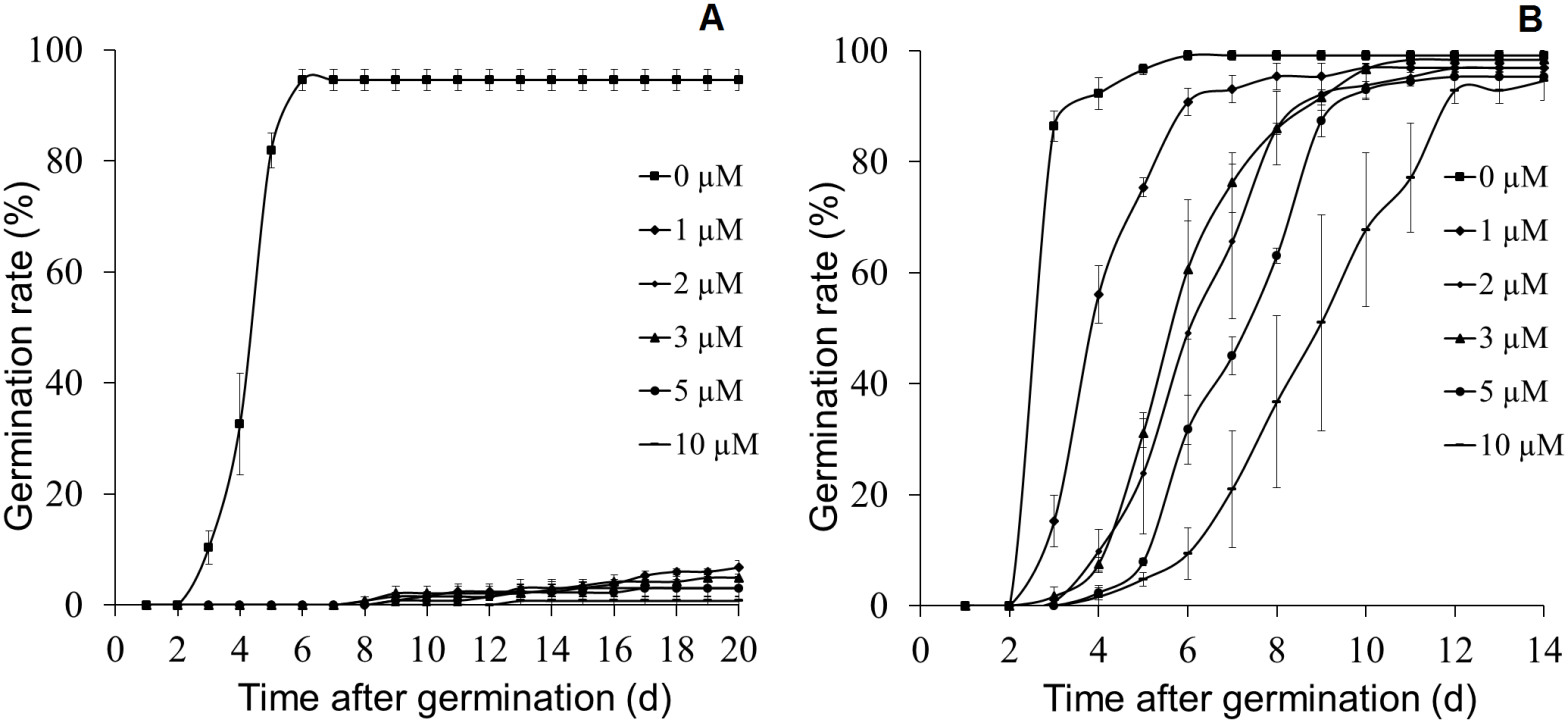
*C*. *australis* seedlings show highly decreased sensitivity to ABA during seed germination. Tomato and *C*. *australis* seeds were sterilized and sown on 1/2 MS medium in the presence of different concentrations of ABA and incubated in a growth chamber (24°C, 16 h light/8 h dark cycle). The germination rates (mean ± SE; N = 30) of tomato (**A**) and *C*. *australis* (**B**) seeds were recorded daily. The experiment was repeated twice with very similar results.

In many plant species, ABA also functions in seed dormancy and germination. Exogenously applied ABA usually causes delayed germination. Thus, we sought to investigate whether *C*. *australis* also would also show decreased ABA sensitivity during germination. Tomato seeds had very poor germination rates on ABA-containing plates: even after 20 days, the germination rates in all treatment groups, including 1 μM, were below 10% (Fig 3A). In contrast, all *C*. *australis* seeds germinated within 14 days, even after being treated with 10 μM of ABA (Fig 3B), indicating that *C*. *australis* seeds are very insensitive to ABA-induced inhibition of seeds germination.

### Dehydration-induced ABA accumulation in *C*. *australis* seedlings

*C*. *australis* seedlings have no roots. To survive the time window of a few days that are needed for finding suitable hosts, they must be able to minimize water loss to counteract drought stress.

**Fig 4:**
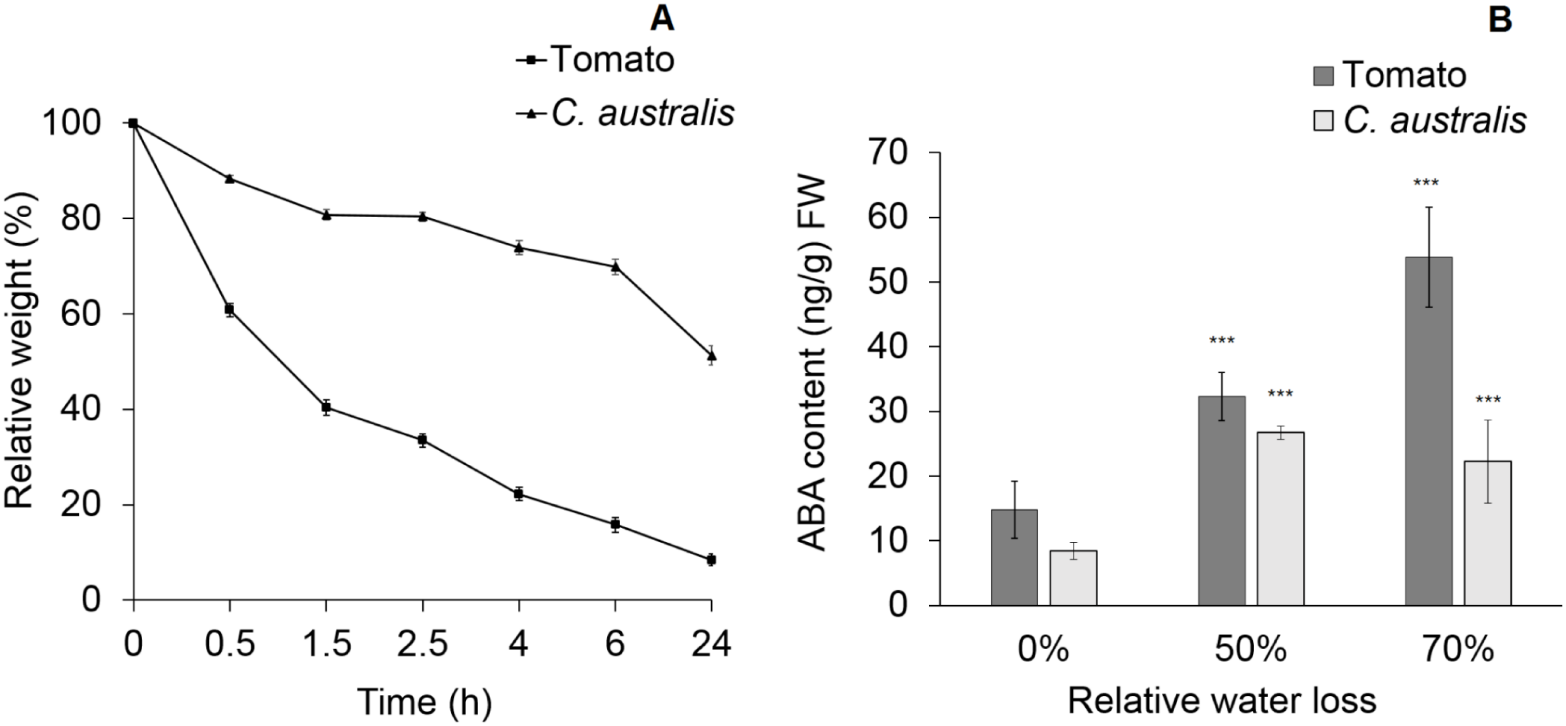
*C*. *australis* seedlings lose water much slower than do tomato seedlings. Tomato and *C*. *australis* seeds were sterilized and sown on 1/2 MS medium and after 5 days of growth, seedlings were placed on plastic foil to dehydrate (air dry). Non-treated seedlings served as controls. (**A**) Dehydration status of tomato and *C*. *australis* seedlings from 0.5 to 24 h (data points were obtained from 0.1 g of seedlings). (**B**) ABA contents in the dehydrated tomato and *C*. *australis* seedlings. Five to 10 seedlings (about 0.1 g) were pooled to form 1 replicate. Values are mean ± SE (N = 5), and asterisks indicate significant differences between the respective air drying-treated and non-treated groups determined by Student’s *t*-test (***, P < 0.001).

Comparison of the water loss of the air-dried *C*. *australis* and tomato seedlings indicated that *C*. *australis* seedlings lost water much more slowly than did tomato seedlings: Tomato seedlings lost 50% of water 1 h after being air-dried, while *C*. *australis* needed 24 h to lose the same percentage of weight (Fig 4A). Given that ABA plays an important role in plant drought-induced responses, the ABA contents in air-dried *C*. *australis* and tomato seedlings were determined (Fig 4B). The ABA contents of tomato seedlings increased 1.18- and 2.64-fold, when the water loss reached 50% and 70%, respectively. Similarly, *C*. *australis* seedlings’ ABA contents elevated 1.17- and 1.64-fold, when water loss was about 50% and 70%, respectively (Fig 4B).

### The ABA receptor genes in *C*. *australis*

**Fig 5:**
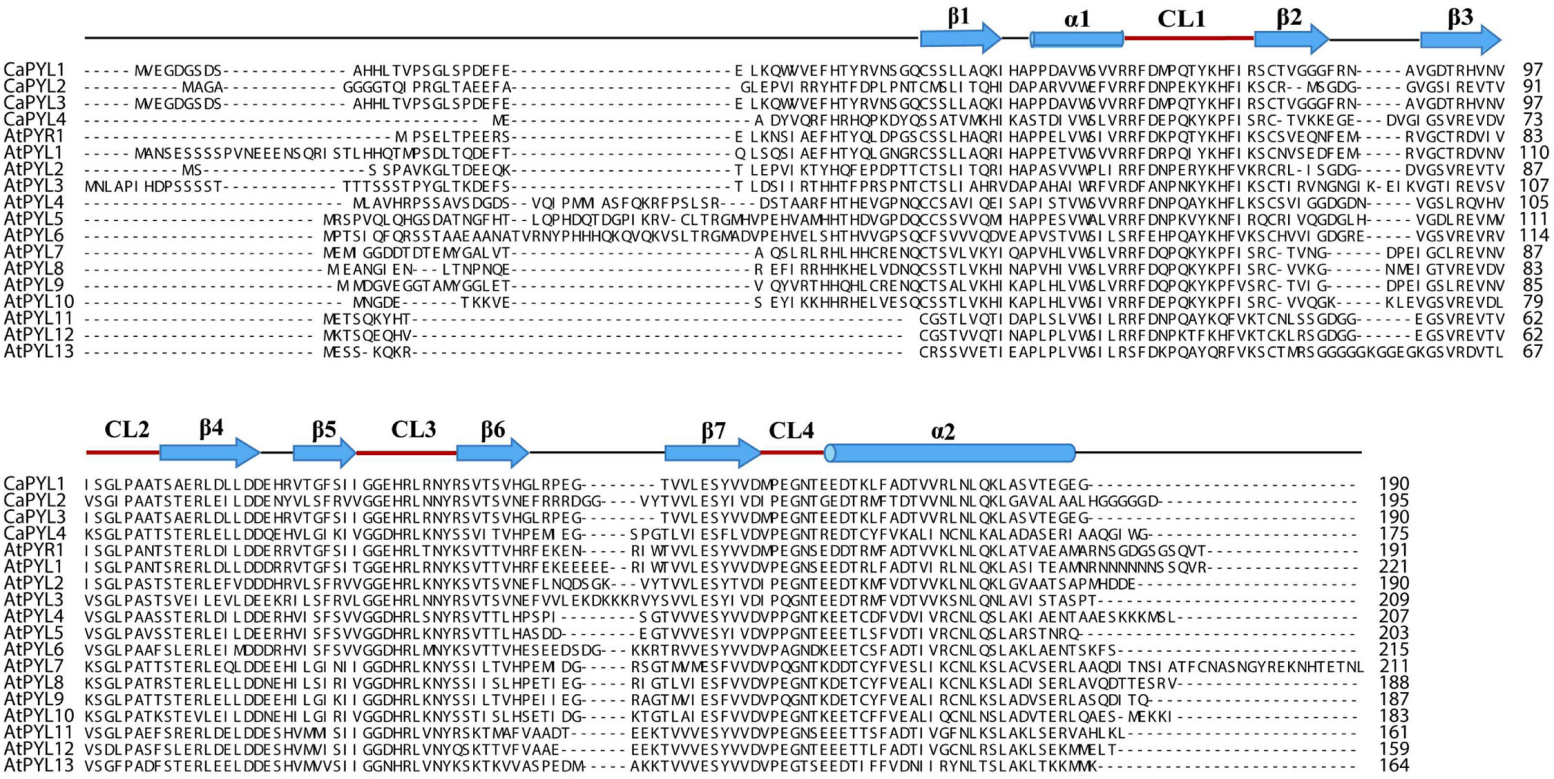
Sequence alignment of *C*. *australis* and *A*. *thaliana* ABA receptors. Secondary structural elements are indicated above the primary sequences. Helices and strands are shown as blue cylinders and arrows, respectively. The 4 conserved loops CL1–CL4 are highlighted by the magenta lines. The 4 ABA receptors of *C*. *australis* are CaPYL1 to CaPYL4, and the 14 ABA receptors of *A*. *thaliana* are AtPYR1 and AtPYL1 to AtPYL13. The alignment was done using ClustalX2.1.

In order to gain insight into the mechanism underlying the highly reduced sensitivity to ABA in *C*. *australis* seedlings, we searched a homemade transcriptome (including seeds, just germinated seeds, seedlings, pre-haustoria, buds, flowers, and capsules) database of *C*. *australis* for ABA receptor genes. Blasting the sequences of the 14 ABA receptors known from Arabidopsis against the *C*. *australis* transcriptome, we obtained 4 ABA receptor candidates that showed e-values < e^-10^ in BLASTP analysis, and they were named CaPYL1 to CaPYL4 (see S1 Table for accession numbers). ABA receptors share a typical domain structure consisting of 7 conserved *β*-sheets and 2 *α*-helices as well as 4 CL loops [26, 29]. Importantly, 2 amino acids, lysine and leucine in the CL1 and CL2 loop, respectively, have been shown to be essential for ABA anchoring in Arabidopsis as PYL13 with deviating sequence is completely irresponsive to ABA [29]. CaPYLs all possessed these key features and were most closely aligned with AtPYR1, AtPYL2, AtPYL5, and AtPYL8 (similarities at the amino acid level were 69, 76, 73, and 71%, respectively). Notably, these *C*. *australis* ABA receptors did not share the AtPYL13-specific abnormality and are thus most likely functional (Fig 5).

**Fig 6:**
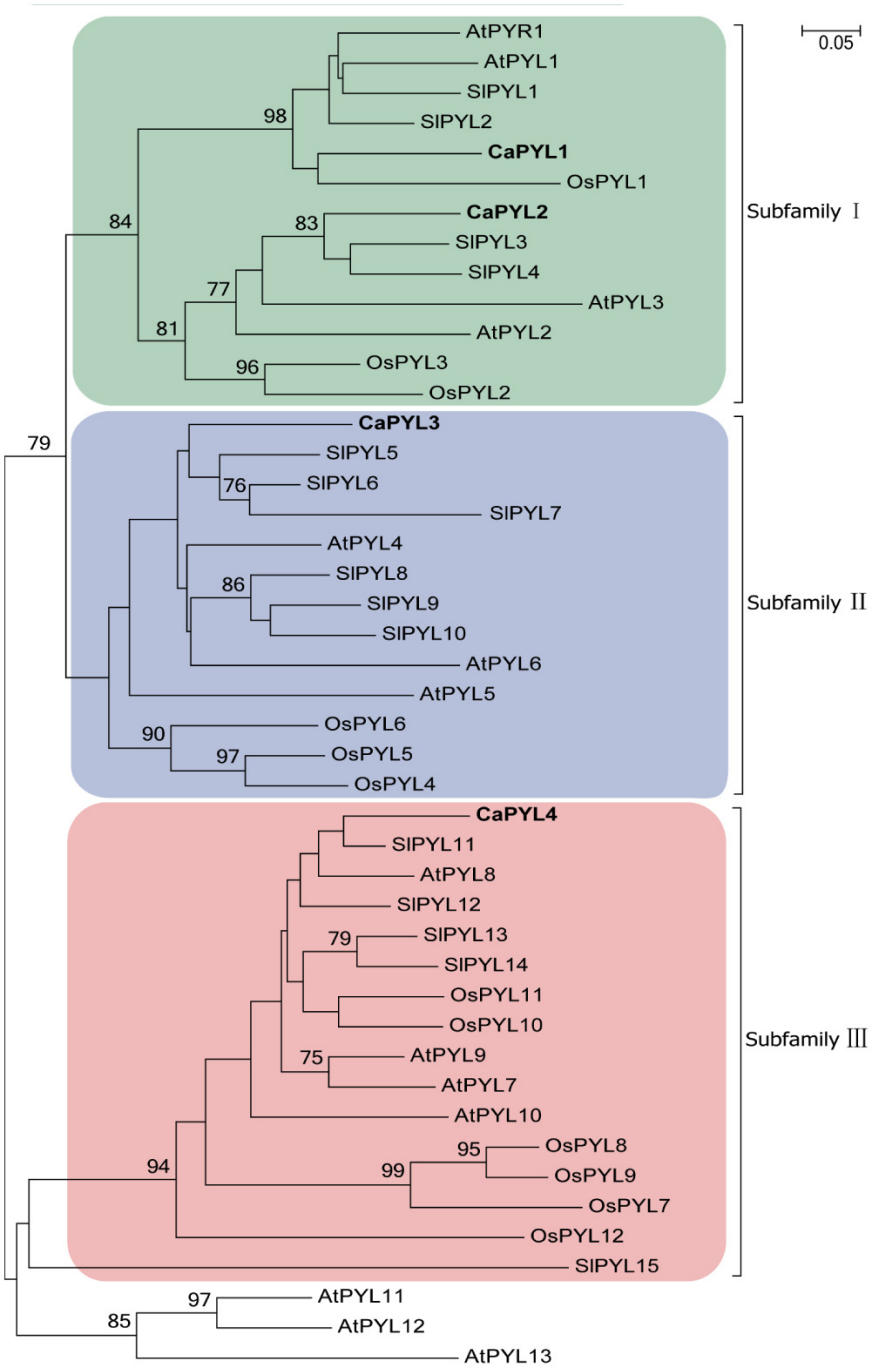
Phylogenetic alignment (phyml/NJ) of the ABA receptor proteins in *Cuscuta australis* (4 receptors), *Arabidopsis thaliana* (14 receptors), *Solanum lycopersicum* (15 receptors), and *Oryza sativa* (12 receptors). The phylogenetic analysis was performed using the neighbor-joining algorithms of MEGA 5.2. The sequences of the 4 CaPYLs are highlighted in bold. Numbers above the branches indicate bootstrap support values of maximum likelihood no less than 75%.

A phylogenetic tree including all ABA receptor proteins in *C*. *australis* (4 receptors), Arabidopsis (14 receptors), tomato (15 receptors), and rice (*Oryza sativa*; 12 receptors) (see S1 Table for accession numbers) was generated to gain insight into the evolution of ABA receptors in *Cuscuta*. Phylogenetic analysis on Arabidopsis had shown that ABA receptors can be grouped into 3 subfamilies [29], and consistently, the four CaPYLs distributed accordingly (Fig 6). CaPYL1 and CaPYL2 were grouped with AtPYR1 and AtPYL1 to AtPYL3 as a dimeric subfamily, CaPYL3 aligned with AtPYL4 to AtPYL6, and CaPYL4 was with the AtPYL7 to AtPYL10 (Fig 6). Notably, consistent with the relatively close relationship between *C*. *australis* and *S*. *lycopersicum* (both in Solanales), CaPYLs grouped closely with tomato ABA receptor proteins, although this gene family in *C*. *australis* was clearly smaller.

## Discussion

Due to their special lifestyles, parasitic plants, especially holoparasitic ones, usually have abnormal morphologies. Root holoparasitic plants may have no leaves, but only stems and flowers; shoot parasitic plants, such as *Cuscuta*, may have no roots and/or leaves. Compared with autotrophic plants, parasitic plants are likely very different in development and stress adaptation. Given the importance of phytohormones in plant development and stress resistance, we examined the responses of *C*. *australis* seedlings to ABA, the most important hormone in drought stress response, and we show that *C*. *australis* seedlings and seeds responded very little to ABA-induced hypocotyl elongation and germination, respectively. Our data suggest that *C*. *australis* has a unique ABA hormonal physiology.

The role of ABA in plant adaptation to drought stress has been intensively studied [30]. Drought stress activates ABA biosynthesis, and the elevated levels of ABA induce stomatal closure, reducing transpirational water loss. Moreover, usually ABA and GA play antagonistic roles in suppressing and promoting germination. We found that ABA strongly suppressed tomato hypocotyl elongation, and when the concentrations were over 1 μM, ABA arrested the growth tomato seedlings. In contrast, *C*. *australis* seedlings only exhibited very little suppression of hypocotyl elongation even when ABA concentration reached 700 μM, a concentration much greater than those in heavily drought-stressed leaves. Similarly, *C*. *australis* seeds germinated well even under high exogenous ABA concentrations.

*C*. *australis* seedlings are basically only thread-like hypocotyls without roots, cotyledons, or true leaves (where stomata are located in seedlings), and their function is mainly to locate hosts and develop haustoria to initiate parasitism. We found that *C*. *australis* seedlings have certain means to minimize water loss (Fig 4), one likely being the greatly reduced number of stomata. After successful parasitization, *Cuscuta* directly draw water from the hosts and even under drought conditions, host plants usually respond strongly with highly elevated ABA levels and closure of stomata to keep relatively stable water contents. Thus, *Cuscuta* may not often be challenged by drought stress, and even under severe drought conditions, ABA signaling might not be able to efficiently help *Cuscuta*, since the already very low transpiration in the stems cannot be further reduced by stomatal regulation. We speculate that ABA signaling in *Cuscuta* might not be as important as in other normal plants, and it may have partially lost its function in *Cuscuta*, and this should be verified further in *Cuscuta* adult plants and in other aspects of ABA-induced responses.

Intriguingly, *C*. *australis* still retained the ability of sensing drought stress and synthesizing ABA (Fig 4B). Qin et al. [31] also found that detached *C*. *reflexa* stems showed increase of ABA contents during dehydration. Similar results were obtained for *C*. *australis* adult stems (data not shown). Thus, *C*. *australis* must still retain the ability to sense drought stress.

It is very likely that most plants have multiple ABA receptors. Our phylogeny analysis indicated that ABA receptors in Arabidopsis, *Cuscuta*, tomato, and rice can be grouped into 3 subfamilies, and each subfamily contained receptor genes from all plant species, suggesting that these subfamilies diverged before the split of monocots and dicots. Even though our transcriptome data were from various *C*. *australis* tissues and developmental stages, we cannot completely rule out that there are more than the four ABA receptor genes in the genome of *C*. *australis*. However, it is likely that *C*. *australis* does not have as many ABA receptor genes as regular plants. One possible explanation is that because of decreased selection pressure, ABA receptor genes have been lost over time in *C*. *australia*. Another scenario, which is less likely, is that the expansion of the ABA receptor gene family happened independently in most plant lineages, and *C*. *australis* (and most likely other *Cuscuta* species) did not expand their ABA receptor gene numbers. It has been speculated that the high diversity of ABA receptor genes in Arabidopsis, tomato, and rice is correlated with their different roles in ABA signaling. The relatively small number of ABA receptors in the *C*. *australis* transcriptome supports the idea that ABA signaling may play a less important role in *C*. *australis* than in normal plants. Research on the function and evolution of ABA signaling in *Cuscuta* deserves further attention. Whether and how *Cuscuta* and other types of parasitic plants have distinct hormonal physiology would be also very interesting to explore.

## Supporting Information

S1 Table Nomenclature and corresponding accession numbers (from NCBI) for the PYR/PYL/RCAR family of ABA receptors of *Cuscuta australis*, *Oryza sativa*, *Arabidopsis thaliana*, and *Solanum lycopersicum*. (PDF)
