# Supplementary material for: The Parasitic Plant Cuscuta australis Is Highly Insensitive to Abscisic Acid-Induced Suppression of Hypocotyl Elongation and Seed Germination
Source: PLoS One. 2015 Aug 10;10(8):e0135197. doi: 10.1371/journal.pone.0135197 (PMC4530876; doi:10.1371/journal.pone.0135197)
Supplement: S1 Table — (PDF) [file pone.0135197.s001.pdf]

S1 Table Nomenclature and corresponding accession numbers (from NCBI) for the PYR/PYL/RCAR family of ABA receptors of *Cuscuta australis*, *Oryza sativa*, *Arabidopsis thaliana*, and *Solanum lycopersicum*

Cuscuta australis		***Oryza sativa***		***Arabidopsis thaliana***		***Solanum lycopersicum***	
Nomenclature	Accession	Nomenclature	Accession	Nomenclature	Accession	Nomenclature	Accession
CaPYL1	KR232648	OsPYL1	NP_001065470.1	PYR1	NP_193521.1	SlPYL1	Solyc06g061180
CaPYL2	KR232649	OsPYL2	NP_001057874.1	PYL1	NP_199491.2	SlPYL2	Solyc08g076960
CaPYL3	KR232650	OsPYL3	NP_001172865.1	PYL2	NP_180174.1	SlPYL3	Solyc12g095970
CaPYL4	KR232651	OsPYL4	BAB68102	PYL3	NP_177443.1	SlPYL4	Solyc08g065410
		OsPYL5	AIX10777.1	PYL4	NP_565887.1	SlPYL5	Solyc03g095780
		OsPYL6	NP_001049838.1	PYL5	NP_196163.1	SlPYL6	Solyc06g050500
		OsPYL7	EEE65809	PYL6	NP_565928.1	SlPYL7	Solyc05g052420
		OsPYL8	NP_001057771.1	PYL7	NP_567208.1	SlPYL8	Solyc10g085310
		OsPYL9	NP_001057772.1	PYL8	NP_200128.1	SlPYL9	Solyc09g015380
		OsPYL10	NP_001046464.1	PYL9	NP_563626.1	SlPYL10	Solyc10g076410
		OsPYL11	NP_001054923.1	PYL10	NP_194521.2	SlPYL11	Solyc01g095700
		OsPYL12	BAD29692.1	PYL11	NP_199398.1	SlPYL12	Solyc03g007310
				PYL12	NP_199399.1	SlPYL13	Solyc08g082180
				PYL13	NP_193597.1	SlPYL14	Solyc12g055990
						SlPYL15	Solyc02g076770
